# Metabolomic Analysis of Carotenoids Biosynthesis by *Sphingopyxis* sp. USTB-05

**DOI:** 10.3390/molecules29174235

**Published:** 2024-09-06

**Authors:** Chao Liu, Qianqian Xu, Yang Liu, Meijie Song, Xiaoyu Cao, Xinyue Du, Hai Yan

**Affiliations:** School of Chemistry and Biological Engineering, University of Science and Technology Beijing, Beijing 100083, China; b20190371@xs.ustb.edu.cn (C.L.); qianqianxu@ustb.edu.cn (Q.X.); liuyang@ustb.edu.cn (Y.L.); meijie_song@126.com (M.S.); d202210452@xs.ustb.edu.cn (X.C.); m202120900@xs.ustb.edu.cn (X.D.)

**Keywords:** *Sphingopyxis* sp. USTB-05, metabolome, carotenoids, zeaxanthin

## Abstract

Carotenoids belonging to the class of tetraterpenoids have extensive applications in medicine, food, nutrition, cosmetics, and feed. Among them, lutein and zeaxanthin can prevent macular degeneration in the elderly, which is very important for protecting vision. Here, we introduce the first metabolomic analysis of *Sphingopyxis* sp. USTB-05, aiming to shed light on the biosynthesis of carotenoids. *Sphingopyxis* sp. USTB-05 has the complete methylerythritol 4-phosphate (MEP) pathway and carotenoid biosynthesis pathway, especially involved in the bioconversion of zeaxanthin, violaxanthin, and astaxanthin. Metabolomic profiling identified seven carotenes and six xanthophylls synthesized by *Sphingopyxis* sp. USTB-05. Zeaxanthin, in particular, was found to be the most abundant, with a content of 37.1 µg/g dry cells. Collectively, the results presented herein greatly enhance our understanding of *Sphingopyxis* sp. USTB-05 in carotenoids biosynthesis, and thus further accelerate its fundamental molecular investigations and biotechnological applications.

## 1. Introduction

Carotenoids are extensively utilized in food, feed, cosmetics, nutrition, and medicine because of their safety and natural properties [1,2]. In nature, carotenoids are present in higher plants, animals, fungi, algae, and bacteria, with more than 800 kinds of natural carotenoids identified [3]. Among them, zeaxanthin is the main pigment in the macular region of the human eye [4,5], but it cannot be synthesized by the human body. Recent studies suggest that a daily intake of 10 mg lutein and 2 mg zeaxanthin can improve visual function and delay the development of age-related macular degeneration (AMD) [6,7]. Currently, commercial zeaxanthin is predominantly sourced from plants and a few microorganisms [8]. The microbial production of carotenoids offers distinct advantages over plant-based methods, notably due to the rapid growth rate and shorter life cycle of microorganisms [9]. Moreover, the downstream extraction process for microbial production is more straightforward, bypassing the need to break down plant cell walls, which can be a complex and costly endeavor. The primary challenge in microbial zeaxanthin production lies in maximizing yield through increased zeaxanthin concentration and microbial biomass [8]. One promising approach is the genetic engineering of microorganisms to produce carotenoids via heterologous expression. Despite this, previous attempts to produce carotenoids using engineered *Escherichia coli* have encountered issues with strain instability and the time-consuming nature of strain construction during the initial phase [10]. This study further improved the carotenoid production of strain USTB-05 by optimizing the cultivation conditions, laying the foundation for achieving efficient carotenoid production in microbial fermentation.

The biosynthesis of carotenoids in eukaryotic organisms has been extensively studied, with their metabolic pathways clearly elucidated in higher plants and microalgae [3,11,12,13]. Specifically, the isopentenyl diphosphate (IPP) or dimethylallyl diphosphate (DMAPP) is synthesized through the mevalonic acid (MVA) pathway using acetyl CoA as the substrate, and the methylerythritol 4-phosphate (MEP) pathway starting from pyruvate and glyceraldehyde 3-phosphate in some higher plants and certain *Streptomyces* [14]. In prokaryotes, the synthesis of carotenoid precursors IPP and DMAPP occurs predominantly via the MEP pathway in bacteria and cyanobacteria [15,16,17]. Interestingly, haloarchaea deviate from this trend, employing the MVA pathway [18,19,20]. Notably, the identification of the carotenoid biosynthetic pathway has been limited to a select few strains in prokaryotes. The existence of the MEP pathway and the MVA pathway in *Sphingopyxis* sp. USTB-05 provides a reference for revealing the molecular mechanism of bacterial diversified carotenoid production.

Numerous studies have documented the remarkable capacity of the Sphingomonadaceae family to degrade environmental pollutants effectively [21,22,23,24]. Among this family, the genus *Sphingopyxis* stands out, yet it has seldom been associated with the synthesis of a diverse spectrum of carotenoids. Additionally, Sphingomonadaceae has earned the distinction of being classified as Generally Recognized As Safe (GRAS). Several of its strains have received regulatory approval from both the United States and the European Union for the production of gellan gum, a versatile ingredient used in the food and cosmetics industries for its gelling, suspending, and stabilizing properties [25].

The objective of our study is to elucidate the types, quantities, and biosynthetic pathways of carotenoids synthesized by *Sphingopyxis* sp. USTB-05 through a comprehensive metabolomics analysis. Our research also endeavors to refine the cultivation process to augment the overall carotenoid yield of this strain. The findings of our study indicate that *Sphingopyxis* sp. USTB-05 possesses significant potential for the industrial-scale production of zeaxanthin, a valuable carotenoid with a range of applications.

## 2. Results

### 2.1. Culture of Strain USTB-05

**Figure 1:**
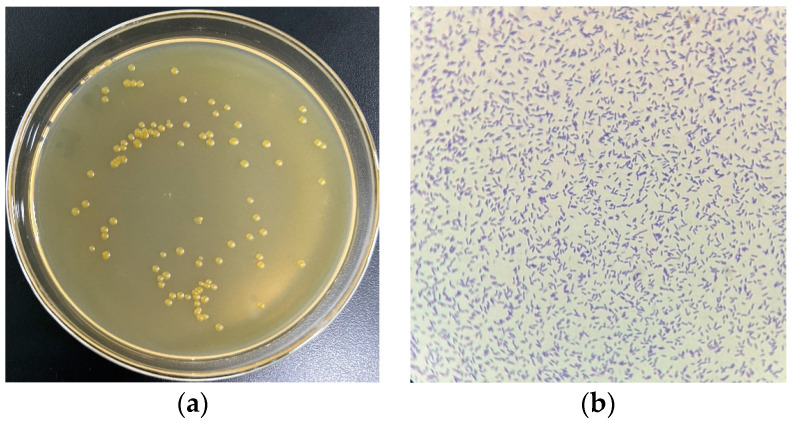
(**a**) Monoclonal colonies and (**b**) crystal violet staining of strain USTB-05 on solid medium.

The monoclonal colonies of USTB-05 were grown on a solid plate, forming a yellow circular colony with smooth and neat edges (Figure 1a). The morphology of USTB-05 was observed under a light microscope with 1000× magnification, appearing short and rod-shaped (Figure 1b).

### 2.2. Determination of Growth Curve of Strain USTB-05

**Figure 2:**
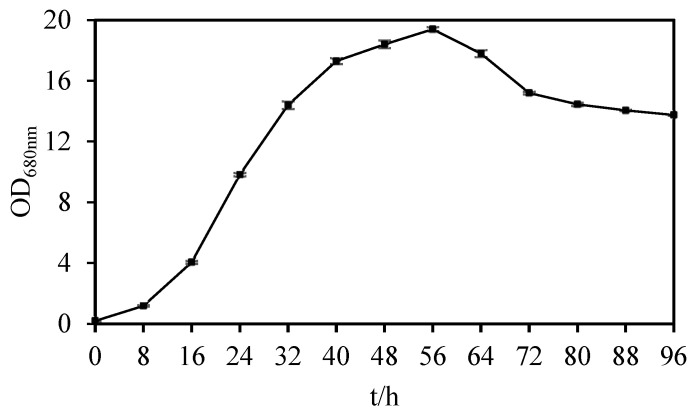
Growth curve of *Sphingopyxis* sp. USTB-05.

*Sphingopyxis* sp. USTB-05 demonstrated its growth dynamics within the culture medium over a period of 4 days (Figure 2). *Sphingopyxis* sp. USTB-05 reached their highest biomass at 56 h, and the OD_680nm_ value was 19.4.

### 2.3. Analysis of Bacterial Composition Metabolites by UHPLC Q-TOF/MS

**Figure 3:**
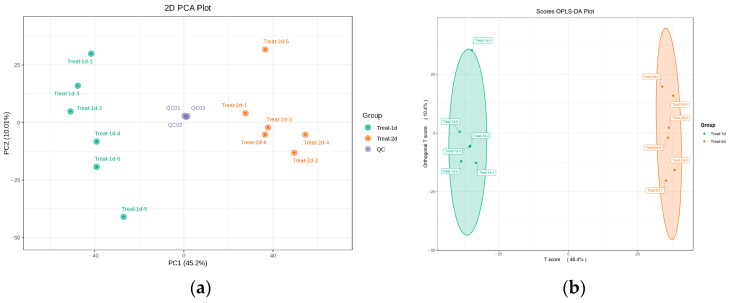
Multivariate statistical analysis of the bacterial composition of strain USTB-05. (**a**) PCA analysis; (**b**) OPLS-DA analysis. Treat-1d: strain USTB-05 at the middle of logarithmic phase (24 h); Treat-2d: strain USTB-05 at the end of logarithmic phase (48 h).

Numerous metabolites were separated throughout the 14 min LC-MS run (Figure S1). QC analyses were tightly clustered in the PCA score plot indicating a reliable metabolomics analysis (Figure 3a). The metabolome profiles of groups cultivated for 24 and 48 h were delineated through a two-dimensional OPLS-DA analysis (Figure 3b). The first two principal components accounted for 45.2% and 10% of the total variance, respectively (R2X = 0.567 for the first two principal components). Two groups were clearly distinguished in the PCA score plot, indicating distinct metabolic patterns among them.

In total, 5881 metabolites were identified (Table 1), encompassing a wide range of biochemical classes such as amino acids, benzene, heterocyclic compounds, organic acids, aldehydes, ketones, esters, alcohols, amines, glycerophospholipids, fatty acids, nucleotides, hormones, flavonoids, terpenoids, alkaloids, carbohydrates, glycerolipids, coenzymes, vitamins, lignans, coumarins, sphingolipids, steroids, tryptamines, cholines, pigments, bile acids, phenolic acids, quinones, and tannins (Table S1). Identification was achieved by matching accurate mass measurements with retention times and referencing metabolomics databases. Specifically, the classification of organic acids and their derivatives included oxal-acetic acid, citric acid, 3-phosphoglycerate, 6-phosphogluconic acid, geranyl diphosphate, mevalonic acid, phosphoenolpyruvate, geranyl diphosphate, 2-C-methyl-D-erythritol 4-phosphate, pyruvate, 1-deoxy-D-xylulose 5-phosphate, and isoprene pyrophosphate, etc. The classification of aldehydes, ketones, and esters featured glyceraldehyde, β-ionone, and 2-hydroxyastaxanthin, etc. The classification of terpenes comprised citrin, violaxanthin, and tetrahydrogeranylgeranyl diphosphate, etc. The classification of carbohydrates was represented by erythrose, erythritol, 2-methyl-D-erythritol 2,4-cyclodiphosphate, D-xylose 5-phosphate, D-fructose 6-phosphate, and trehalose 6-phosphate, etc. (Table S2). Mevalonic acid, a key compound in the biosynthesis of terpenoids by the MVA pathway, has been well-documented in *Streptomyces* [14]. Similarly, 1-deoxy-D-xylulose 5-phosphate, pivotal in the synthesis of carotenoids through the MEP pathway, has been elucidated in the marine bacterium *Echinicola marina* [26]. Additionally, β-ionone stands out as a distinctive functional group of xanthophyll compounds.

### 2.4. Identification and Content Alterations of Carotenoids during Cultivation

During the 11 min LC-MS run, a diverse array of metabolites was isolated from the detected samples, with all carotenoids being identified in positive ion mode (Figure S2). A total of 13 distinct carotenoid types were identified in strain USTB-05, encompassing seven carotenes and six xanthophylls (Table 2). Carotenes included α-carotene, β-carotene, phytoene, ε-carotene, phytofluene, γ-carotene, and lycopene. Among them, metabolites exceeding a concentration of 0.01 µg/g are detailed in Table 3. The average content of phytoene was the highest, at 2.32 µg/g, in the middle of logarithmic phase, and the average content of phytofluene was the highest, at 0.42 µg/g, at the end of the logarithmic phase. Concluding the logarithmic phase, the expression levels of α-carotene, β-carotene, phytoene, and phytofluene were all downregulated. Notably, β-carotene and phytoene experienced significant reductions, amounting to 84.46% and 84.85%, respectively. Xanthophylls included echinenone, zeaxanthin, violaxanthin, β-cryptoxanthin, β-citraurin, and astaxanthin. Among them, metabolites exceeding a concentration of 0.01 µg/g are detailed in Table 3. The average content of zeaxanthin was the highest at the middle of the logarithmic phase and the end of the logarithmic phase, with 37.06 µg/g and 35.25 µg/g, respectively. Zeaxanthin was downregulated at the end of the logarithmic phase with a decrease of 4.9%.

### 2.5. Elucidating the Biosynthetic Pathway of Carotenoids in Sphingopyxis sp. USTB-05

**Figure 4:**
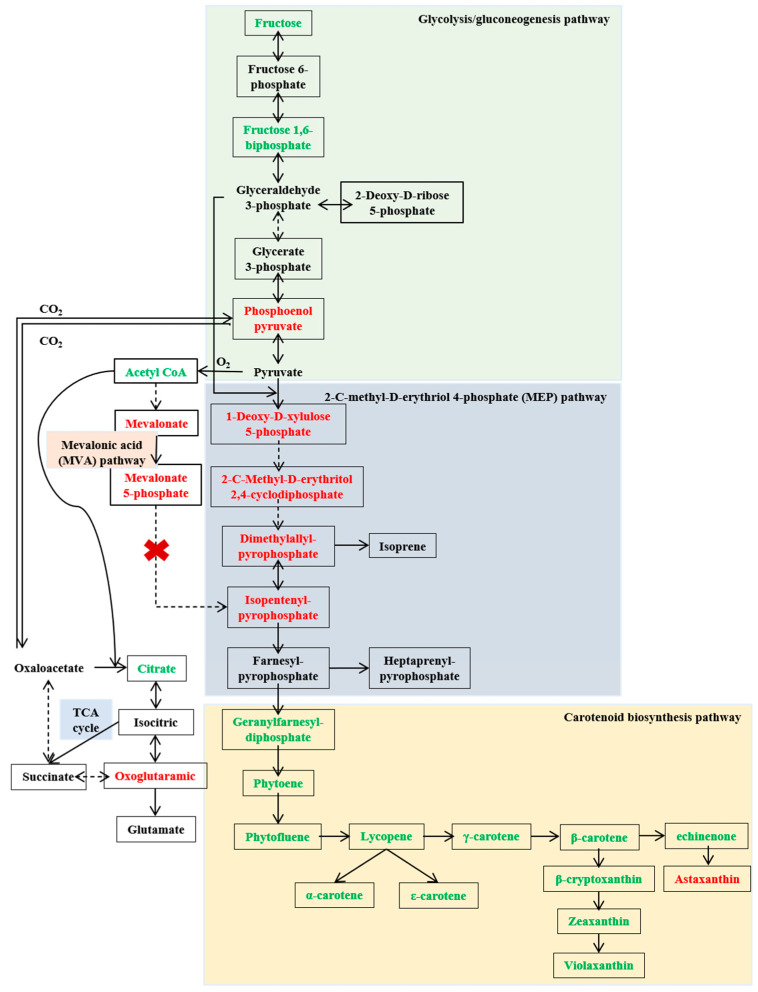
Undetected key metabolites are shown without a box. The dashed line indicates the presence of multi-step reactions. The red font represents cultivation for 48 h to upregulate metabolites. The green font represents cultivation for 48 h to downregulate metabolites.

The analysis of differential metabolites in the glycolysis pathways revealed that *Sphingopyxis* sp. USTB-05 produced glyceraldehyde 3-phosphate and pyruvate through the glycolysis pathway, which are key precursors for the generation of 1-deoxy-D-xylulose 5-phosphate, essential for terpenoid compound synthesis via the MEP pathway (Figure 4). The intermediate metabolite fructose 1,6-diphosphate in the glycolysis pathway was significantly downregulated, while phosphoenolpyruvate exhibited a significant upregulation during the logarithmic phase. Additionally, oxaloacetic acid was catalyzed to generate phosphoenolpyruvate in the tricarboxylic acid cycle of *Sphingopyxis* sp. USTB-05.

Differential metabolite analysis between the MEP and MVA pathways indicated that *Sphingopyxis* sp. USTB-05 synthesized Geranylfarnesyldiphosphate (GFDP) through the MEP pathway, with 1-deoxy-D-xylulose 5-phosphate, 2-C-methyl-D-erythritol 2,4-cyclodiphosphate, dimethylallyl pyrophosphate, and isopentenyl pyrophosphate significantly upregulated. Noticeably, the key intermediate metabolite mevalonate-5-pyrophosphate in the MVA pathway was undetected in *Sphingopyxis* sp. USTB-05. Combined with genomic analysis, no genes associated with phosphomevalonate kinase and mevalonate 5-pyrophosphate decarboxylase were found, indicating the absence of the MVA pathway in *Sphingopyxis* sp. USTB-05 [27].

In the biosynthesis process from GFDP to xanthophyll compounds, *Sphingopyxis* sp. USTB-05 utilizes GFDP as a substrate. Through the catalytic action of phytoene synthase, GFDP undergoes condensation to form phytoene. Phytoene is further dehydrogenated by dehydrogenase to generate phytofluene, which is subsequently converted into lycopene. The cyclization of lycopene marks a critical juncture in carotenoid synthesis and metabolism, leading to the formation of α-carotene, ε-carotene, and β-carotene under the catalysis of various lycopene cyclases. β-carotene is converted into echinenone by β-carotene 4-ketolase and then into astaxanthin through a two-step process involving β-carotene 3-hydroxylase and zeaxanthin 4-ketohydrolase. β-carotene can also be transformed into β-cryptoxanthin by β-carotene 3-hydroxylase, which further catalyzes the production of zeaxanthin. Finally, violaxanthin arises from the cyclization reaction catalyzed by zeaxanthin epoxidase.

### 2.6. Optimization of Culture Conditions of Sphingopyxis sp. USTB-05

**Figure 5:**
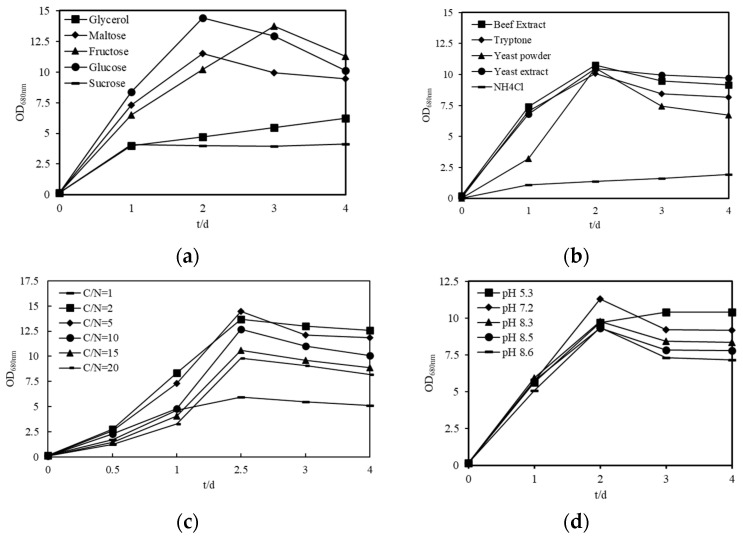
Optimization of culture conditions for enhanced *Sphingopyxis* sp. USTB-05 biomass. The strain USTB-05 was cultivated in media with different (**a**) carbon sources, (**b**) nitrogen sources, (**c**) C/N ratios, and (**d**) pH values.

To enhance the carotenoid yield in *Sphingopyxis* sp. USTB-05, a comprehensive optimization of the culture conditions was undertaken. This optimization focused on various parameters, including carbon sources, nitrogen sources, C/N ratio, and pH. The biomass of the strain USTB-05 cultivated with various media is shown in Figure 5. Among the carbon sources evaluated, D-glucose, D-maltose, and D-fructose proved to be the most favorable for cellular proliferation. The maximum biomass, reaching 14.4, was achieved with cells cultured in a medium containing 20 g/L D-glucose over 48 h (Figure 5a). In the assessment of nitrogen sources, beef extract, tryptone, yeast powder, and yeast extract proved to be the best nitrogen sources for cellular proliferation. The results indicate a preference for organic nitrogen sources in promoting the growth of strain USTB-05 (Figure 5b). Interestingly, an increase in the concentration of beef extract corresponded with an increase in biomass, with the optimum observed at a C/N ratio of 5 (Figure 5c). Furthermore, the optimal pH for the growth of strain USTB-05 was determined to be 7.2, under which conditions the strain exhibited the most robust growth (Figure 5d).

### 2.7. Determination of Carotenoid Content in Fermentation Broth of Strain USTB-05 at Different Growth Stages

**Figure 6:**
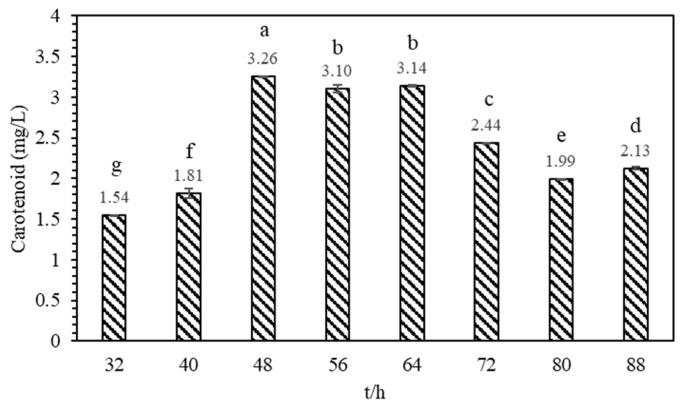
Determination of carotenoid content in different growth stages of *Sphingopyxis* sp. USTB-05. Mean ± SD. The different letters on the data indicate the degree of difference (*p* < 0.05).

To investigate the changes in total carotenoid content produced by *Sphingopyxis* sp. USTB-05 at different growth stages, eight time points were selected for sampling and measurement. At 48 h of cultivation, coinciding with the end of the logarithmic phase, the highest content of carotenoids in the bacterial solution of strain USTB-05 reached 3.26 mg/L (Figure 6). The results demonstrate that the proliferation of *Sphingopyxis* sp. USTB-05 is concurrent with the biosynthesis of carotenoids. However, the efficiency of this carotenoid production is subject to variation across the different phases of bacterial growth. Notably, the most substantial accumulation of carotenoids was observed at 48 h cultivation. At the same time, it was observed that the carotenoid content per unit of fermentation broth significantly decreased in the decline phase.

## 3. Discussion

*Sphingopyxis*, a distinctive genus of the Sphingomonadaceae family, is a ubiquitous bacterium with a wide of range ecological habitats, from the landfills of terrestrial habitats to lakes and marine environments. *Sphingobium*, *Novosphingobium*, *Sphingopyxis* and *Blastobacter ursincola* are recognized as junior synonyms of species within the genus *Sphingomonas* [28]. Colonies are yellow, round, convex, smooth, shiny, moist and sticky, with neat edges. Cells are aerobic, short rod-shaped, Gram-negative, without spores, and with polar flagella on one side. The main respiratory quinone is coenzyme Q10 in the cytoplasmic membrane. The only glycolipid is glycosphingolipid in the cell membrane. The fatty acid composition contains 2-hydroxy fatty acids and no 3-hydroxy fatty acids. The genomic GC content of *Sphingomonas* ranges from 61.7% to 67.2% [29,30].

### 3.1. The Function of Sphingomonas

*Sphingomonas* has extremely high application value in food, medicine, environmental remediation, and agricultural development. (1) The welan gum it produces is a natural microbial polysaccharide in food and medicine. The welan gum is proven as safe, non-toxic, and biodegradable in countries such as the United States and Japan [31]. Additionally, welan gum is also used in the formulation of emulsion and suspension drugs. (2) *Sphingomonas* exhibits superior biodegradation ability for aromatic compounds and certain heavy metal compounds in terms of environmental remediation [32]. (3) Certain strains of *Sphingomonas* have the effects of promoting plant growth and improving plant stress resistance, and represent promising candidates for crop growth promotion in agricultural development. They offer substantial potential for decreasing the reliance on fertilizers and pesticides, thereby alleviating agricultural challenges in impoverished regions. Although numerous studies have introduced its biotechnological potential in the industrial production of several bioactive compounds, there is limited understanding of the genetic and metabolic basis of the key metabolic pathways necessary to reveal the carotenoids of *Sphingomonas*. This study utilizes further metabolomics analysis based on LC-MS technology, which provides comprehensive information to reveal the metabolic pathways of carotenoids in *Sphingopyxis* sp. USTB-05.

### 3.2. The Antioxidant Properties of Carotenoids

Cyanobacterial hepatotoxins, such as microcystins (MCs) and nodularins (NODs), are potent toxic metabolites generated by cyanobacteria during water blooms. These substances are not only produced in the greatest quantities, but also exhibit the broadest distribution and pose the most significant threat to environmental and public health. *Sphingopyxis* sp. USTB-05 isolated from the sediment of Dian chi Lake can efficiently biodegrade microcystins. The strain and its crude enzymes are capable of completely degrading microcystin-RR (MC-RR) from an initial concentration of 42.3 mg/L within 36 h and 10 h [33]. The first important gene (USTB-05-A, 1008 bp) involved in the biodegradation of MC-RR, microcystins LR, YR (MC-LR, MC-YR), was cloned from *Sphingopyxis* sp. USTB-05 [34] and successfully expressed in *Escherichia coli* BL21 using the pGEX4T-1 expression vector. With a total protein concentration of 350 mg/L, the initial concentrations of 40 mg/L for MC-RR or MC-LR, and 14.8 mg/L of MC-YR, were completely biodegraded within 0.25 h, 1 h, and 10 h, respectively [35,36,37]. The second enzyme encoded by USTB-05-B was verified by heterologous expression in *Escherichia coli* to catalyze linear MC-RR into a tetrapeptide by breaking the Ala–Arg bond. The third enzyme encoded by USTB-05-C can cleave the Adda-Glu peptide bond of linear MC-RR and the tetrapeptide of Adda-Glu-Mdha-Ala, producing Adda as their common product [38]. Microcystinase (Mlr A) catalyzes the first and most important biodegradation step of microcystins produced and released by cyanobacterial cells. Subsequently, a site-directed mutant of MlrA is constructed for verification. The results reveal that MlrA is not a metalloprotease, but a glutamate protease classifying within the type II CAAX prenyl endopeptidase family [39]. Cyclic MCs are converted into linear MCs, tetrapeptides, and Adda through the *mlr* degradation pathway by USTB-05-A enzyme, USTB-05-B enzyme, and USTB-05-C enzyme [37]. Alternatively, USTB-05-A enzyme and USTB-05-C enzyme can convert NOD into linear NOD and Adda [40]. After Adda is degraded to phenylacetic acid (PAA), it is converted to PAA-CoA by PAAase. PAA-CoA is degraded to acetyl coenzyme A (Acetyl-CoA) through the PAA degradation pathway. Finally, Acetyl-CoA enters the tricarboxylic acid cycle and degrades to CO_2_ [41]. *Sphingopyxis* sp. USTB-05 can degrade hepatotoxins, a process that invariably generates reactive oxygen species and other oxidants. Reactive oxygen or other oxidants can damage cellular components such as DNA, proteins, and membranes. Consequently, the carotenoids synthesized by *Sphingopyxis* sp. USTB-05 play a crucial role in shielding the cells from oxidative stress-induced damage. Similar protective mechanisms also exist in other *Sphingomonas* strains. For instance, during the biodegradation of heterocyclics (carbazole, benzothiophene and dibenzothiophene), the amount of zeaxanthin and hydrogen peroxide produced by *Sphingobium yanoikuyae* sp. XLDN2-5 increases significantly at the same time [42].

### 3.3. The Types and Contents of Carotenoids

*Sphingopyxis* sp. USTB-05, as a promising non-conventional bacterium, offer potential advantages to the industrial production of carotenoids. The potential of *Sphingopyxis* sp. USTB-05 stands out significantly in comparison to algae and plants, particularly due to its shorter life cycle, higher biomass production, and its remarkable ability to flourish on a variety of cost-effective agro-industrial materials. *Sphingopyxis* sp. USTB-05, a hyper-producer of carotenoids, principally synthesizes a mixture of carotenoids, including zeaxanthin (37.1 µg/g), phytoene (2.3 µg/g), phytofluene (0.5 µg/g), α-carotene (0.3 µg/g), β-carotene (0.1 µg/g), and β-cryptoxanthin (0.1 µg/g). In terms of carotenoid production in eukaryotes, the chlorella is notable for its high lutein content. The mutant *chlorella zofingiensis* CZ-bkt1 has a lutein content of up to 13.81 mg/g, which is 16 times higher than that of marigold [43]. Through enzyme molecular modification, *Yarrowia lipolytica* can produce 39.5 g/L β-carotene with a productivity of 0.165 g/L/h (1441 times higher than the initial strain), which is the highest level of β-carotene production reported recently [44]. *Xanthophyllomyces dendrorhous* has been instrumental in large-scale production, with the highest recorded content reaching 3 mg/g on dry cells [45]. Utilizing ARTP mutagenesis, the astaxanthin yield in brewing yeast has seen a significant increase, from 5.5 mg/g to 10.1 mg/g. As for the production of carotenoid by prokaryotes, the optimized cultivation of *Rhodococcus opacus* PD630 has yielded a β-carotene production of 0.7 mg/L [46]. *Sphingomonas* can synthesize a variety of carotenoids, but the relative proportions of lycopene, carotene, and xanthophyll in *Sphingomonas* are not constant and may be affected by a variety of culture conditions and different strain specificities. More hydrogen lycopene belongs to carotenoids, tetraterpenoid compounds that are abundant in tomatoes and tomato products and are a potent antioxidant and non-provitamin A carotenoids. More hydrogen lycopene has the function of improving cancer onset, diabetes, cardiac complications, oxidative stress-mediated dysfunction, inflammation, and skin, bone, liver, neurological and reproductive diseases [47]. The antioxidant properties of more hydrogen lycopene can effectively prevent the oxidation of fats and their associated products, thereby enhancing the shelf life and quality of these commodities [48]. Lycopene has been widely studied for Diabetes Mellitus Type 2 (T2DM) treatment efficiency [49]. An assortment of xanthophyll varieties includes lutein, zeaxanthin, β-cryptoxanthin, astaxanthin, and violaxanthin. The effects of lutein and zeaxanthin have been studied for a variety of diseases, including neurological, ophthalmic, oral, allergic, and immune diseases [50]. β-cryptoxanthin is a promising candidate drug for preventing bone loss by promoting osteoblastic bone formation and inhibiting osteoclastic bone resorption [50]. Although more hydrogen lycopene, carotene, and xanthophylls have broad and important application potential, the current yields from most strains are insufficient for satisfying the demands of industrial-scale production. Current research focuses on improving the production of carotenoids through fermentation process optimization, mutagenesis breeding and strain domestication. Using genetic engineering strategies to modify the carotene production pathway offers an alternative way to further increase the production of carotenoids [51]. Nevertheless, the complete biosynthetic pathway of xanthophylls in *Sphingomonas* remains to be verified, especially the biotransformation from GFDP to violaxanthin and astaxanthin. Utilizing the metabolomic analyses based on the LC-MS technologies, the present study provides a comprehensive overview of the proposed metabolic pathways involved in the carotenoid biosynthesis of *Sphingopyxis* sp. USTB-05.

### 3.4. The Biosynthetic Pathway of Carotenoids

A rough biosynthetic pathway for carotenoids in *Sphingobium* sp. KIB has been proposed previously [8], which encompasses three principal stages: (1) pyruvate and glyceraldehyde-3-phosphate biosynthesis, (2) terpenoid skeleton biosynthesis, and (3) carotenoid biosynthesis. In the first stage, the initial phase of this pathway involves variability in the synthesis routes for pyruvate and glyceraldehyde-3-phosphate in different *Sphingomonas*. For instance, previous studies concluded that glyceraldehyde 3-phosphate and pyruvate are synthesized through the Entner–Doudoroff (ED) pathway in *Sphingobium* sp. KIB [8], while *Sphingopyxis* sp. USTB-05 utilizes the glycolysis pathway. Our study, employing non-targeted metabolomics analysis, has corroborated the synthesis pathways of glyceraldehyde-3-phosphate and pyruvate in *Sphingopyxis* sp. USTB-05, aligning with the predictions derived from previous genomic analyses of the strain [27]. In the second stage, bacterial isoprenoids are synthesized from ubiquitous isopentenyl units, isopentenyl diphosphate (IPP) and its isomer dimethylallyl diphosphate (DMAPP), via the 2-C-methyl-D-erythritol 4-phosphate (MEP) pathway [52]. A case in point is *Azospirillum brasilense* sp. Cd, which synthesizes the isopentenyl unit of squalene (C30) precursor through the MEP pathway [53]. Similarly, *Altererythrobacter ishigakiensis* sp. NBRC107699 produces IPP and DMAPP of astaxanthin precursors via the MEP pathway [54]. In this study, *Sphingopyxis* sp. USTB-05 synthesizes isopentenyl units, which are precursors to zeaxanthin, violaxanthin, and astaxanthin, employing the MEP pathway [27]. The MVA pathway is another widely present pathway for synthesizing IPP and DMAPP, which serve as precursors for all known carotenoids. Haloarchaea in the archaea group synthesize carotenoids through the MVA pathway [18]. In certain higher plants and *Streptomyces*, IPP and DMAPP can be synthesized by the MVA pathway utilizing acetyl-CoA as the substrate, as well as through the MEP pathway utilizing pyruvate and 3-phosphoglyceraldehyde as the substrate [14]. It is worth noting that the intermediate metabolites such as mevalonic acid and 5-phosphomevalonic acid were detected, despite the absence of a complete MVA pathway in *Sphingopyxis* sp. USTB-05. By employing *Sphingopyxis* sp. USTB-05 as the host organism and engineering it to incorporate a comprehensive MVA pathway, it will amplify the strain’s capacity for carotenoid biosynthesis. In the third stage, six enzymes (CrtE, CrtB, CrtI, CrtY, CrtZ, and CrtG) are involved in the continuous condensation process of IPP and DMAPP in *Sphingomonas* sp. ATCC 55669 and *Sphingobium* sp. KIB [8,55]. In a contrasting process, six enzymes (CrtB, CrtIa, CrtIb, CrtL-b, CrtZ, and CrtR-b) are involved in the continuous condensation process of GGPP in *Sphingomonas morindae* sp. NBD5 and *Sphingopyxis* sp. USTB-05 [27]. *Sphingobium* sp. KIB accumulates nostoxanthin rather than zeaxanthin, and the key enzyme catalyzing the conversion of intermediate metabolite zeaxanthin to nostoxanthin is CrtG. [8]. Noticeably, *Sphingopyxis* sp. USTB-05 can naturally accumulate higher levels of zeaxanthin. Through untargeted metabolomics analyses of bacterial components, it was predicted that *Sphingopyxis* sp. USTB-05 can also produce astaxanthin from β-carotene. Therefore, the genetic diversity among *Sphingomonas* endows them with distinct biosynthetic pathways for carotenoid production, leading to variations in both the types and quantities of carotenoids yielded by each strain.

Based on qualitative research into carotenoid standards, we discovered that *Sphingopyxis* sp. USTB-05 is capable of synthesizing violoxanthin and astaxanthin. Violaxanthin, a xanthophyll derived from plants, is a pivotal precursor of abscisic acid, fucoxanthin, capsorubin and β-damascenone, which shows extensive commercial value in food, agriculture, cosmetics, and industries [56]. However, most plant xanthophylls have naturally low concentrations in plants, significantly limiting their commercial applications [57]. Astaxanthin, a ketocarotenoid with exceptional antioxidant properties, demonstrates higher antioxidant activity compared to many other carotenoids. This makes it a valuable compound in cosmetics, aquaculture, nutraceuticals, therapeutics, and pharmaceuticals [58]. More and more evidence suggest that astaxanthin has important nutritional and health benefits in the field of dermatology. Specifically, astaxanthin has protective effects against UV-induced skin damage, anti-allergic properties in treating contact dermatitis, a role in preventing oral lichen planus, and potential utility in mitigating the progression of early-stage cancers [59]. The synthesis process of violaxanthin and astaxanthin in other bacteria is as follows: Zeaxanthin is catalyzed by zeaxanthin epoxidase ZEP through a cyclization reaction to form violaxanthin. β-carotene is catalyzed by β-carotene 4-keto enzyme CrtW to produce echinenone, which is catalyzed by β-carotene 3-hydroxylase CrtZ and zeaxanthin 4-keto hydrolase CrtO to produce astaxanthin. Elucidating the complete carotenoid biosynthetic pathway will provide key genetic targets for regulating the metabolic flux of zeaxanthin, violaxanthin and astaxanthin in *Sphingopyxis* sp. USTB-05. This work may be used for the construction and cultivation of industrial platform strains for carotenoid production, especially zeaxanthin, violaxanthin and astaxanthin.

In this study, the absence of the carotenoid MVA pathway in strain USTB-05 is inferred primarily from genomic and metabolomic data analyses. Further confirmation is essential, which can be achieved through isotope labeling, gene knockout, and vitro enzyme assays. This study achieved the cultivation of high concentrations of strain USTB-05 through strain cultivation optimization experiments. However, compared with some existing industrial production strains, the yield remains relatively modest and warrants further enhancement. Based on the elucidated pathway of carotenoid synthesis metabolism, the metabolic engineering strategies that can be implemented primarily encompass the optimization of metabolic pathways and fluxes, modular enzymes assembly, optimization of promoters and copy numbers, cofactor engineering, and the compartmentalization of carotenoid synthesis regions.

## 4. Materials and Methods

### 4.1. Bacterial Strain and Culture Conditions

*Sphingopyxis* sp. USTB-05 was isolated from the sediment in Lake Dianchi (Kunming City, China) for biodegrading cyanobacterial hepatotoxin [33]. *Sphingopyxis* sp. USTB-05 was cultured in nutrient broth medium (NBM) at 30 °C with shaking (200 rpm). NBM medium contains 15 g of beef extract, 20 g of glucose, 0.5 g of MgSO_4_·7H_2_O, 1 g of KH_2_PO_4_, 1.2 g of Na_2_CO_3_, and 0.02 g of CaCl_2_ per liter. The pH value was adjusted to 7.0 with HCl or NaOH. For the testing of the carbon sources or nitrogen source utilization, the glucose or beef extract was replaced with 20 g/L of other carbon sources or 15 g/L of other nitrogen sources. Different concentrations of beef extract were added to the inorganic salt culture medium as nitrogen sources, with carbon to nitrogen ratios of 1, 2, 5, 10, 15, and 20, respectively. The UV–visible spectrophotometer optical density at 680 nm was used to determine the cell mass.

### 4.2. Bacterial Sample Treatment

A monoclonal colony was initially inoculated into 10 mL NBM medium for 5 days at 30 ℃, with the shake rate of 200 rpm. Exponential-phase cells were inoculated into 20 mL of NBM medium at a 1% inoculation rate in 100 mL erlenmeyer flasks. Six parallel samples were taken at 24 and 48 h of cultivation for metabolomics analysis. Samples were mixed and centrifuged at 6000 r/min for 10 min at 4 °C. For this, 20 mL of culture was washed twice with 50 mmol phosphate buffer (pH 7.0) and the centrifugation operation was repeated. Centrifuged cells were dried to a constant weight by freeze-drying.

Each sample was finely ground using a grinder (MM400, Verder Shanghai Instrument and Equipment Co., Ltd., Shanghai, China). Each sample (20 mg) was placed in a 1.5 mL centrifuge tube, with the addition of 400 μL of 70% methanol aqueous solution containing internal standard. The mixture was vortexed for 3 min and stored in a refrigerator at 4 °C for 30 min. The extraction solution was centrifuged at 12,000 r/min for 10 min at low temperature. After repeating the operation once, the extraction solution (200 μL) was taken for high-performance liquid chromatography analysis.

### 4.3. Untargeted Metabolomics Analysis

Bacterial samples were analyzed using a SHIMADZU Ultra Performance Liquid Chromatography (UPLC) infinity system equipped with a waters ACQUITY UPLC BEH C18 1.8 μm column (2.1 × 100 mm) with a constant column temperature of 40 °C (Shimadzu, Tokyo, Japan). Binary mobile phases were used for elution through an online mixing of solvent A and solvent B, where solvent A was an aqueous solution containing 0.1% (*v*/*v*) formic acid and solvent B was an acetonitrile solution containing 0.1% (*v*/*v*) formic acid. The linear gradient program was as follows: 0 min, 5% B; 11 min, 90% B; 12 min, 90% B; 12.1 min, 5% B; 14 min, 5% B. The total elapsed time required for a given chromatographic analysis was thus 14 min. The flow rate was set at 0.4 mL/min. The injection volume was 2 μL. The effluent from the column was directed to a dual jet stream electrospray ionization (ESI) source interfaced to the Q-TOF mass spectrometer with the following parameters: the capillary voltage was set as 5500 V (Positive ion mode)/4500 V (Negative ion mode); the temperature was set at 550 °C (Positive ion mode)/450 °C (Negative ion mode); the nebulizer pressure was 35 psi; the mass scan range of 50–1000 Da was applied for first-order mass spectrum analysis; the mass scan range of 25–1000 Da was applied for second-order mass spectrum analysis.

### 4.4. Extraction and Measurement of CAROTENOIDS

#### 4.4.1. Determination of Carotenoid in Bacterial Cells

Three parallel samples were collected at 24 and 48 h of cultivation for the measurement of carotenoids. Each sample was ground for 1 min to a fine powder using a grinder (MM400, Verder Shanghai Instrument and Equipment Co., Ltd., China) operated at 30 Hz. Each freeze-dried sample (50 mg) was mixed with 0.5 mL of a hexane/acetone/ethanol solution (1:1:1, *v*/*v*/*v*) containing 0.01% 2-tert-butyl-4-methylphenol (BHT). This mixture was placed in an ice bath and subjected to ultrasonication for 5 min to facilitate extraction. After whirling for 10 min at room temperature, the supernatant was separated by centrifugation at 12,000 r/min for 5 min at 4 °C. This extraction process was repeated twice for each sample. Then, by mixing, the supernatant of the same sample was concentrated and dissolved in a 100 μL methanol/methyl tert-butyl ether solution (50%/50%, *v*/*v*). Finally, the sample solutions were filtered through a 0.22 μm membrane and stored in brown injection bottles for LC-MS analysis.

Carotenoids were analyzed and quantitated using a SCIEX Ultra Performance Liquid Chromatography (UPLC) ExionLC™ AD infinity system equipped with a YMC Carotenoid C30 3 μm column (2.0 × 100 mm) with a constant column temperature of 28 °C (Sciex, Framingham, MA, USA). The mobile phase was composed of solvent A (25% methanol and 75% acetonitrile, containing 0.1% formic acid and 0.01% 2,6-di-tert-butyl-4-methylphenol) and solvent B (tert-butyl methyl ether solution containing 0.01% 2,6-di-tert-butyl-4-methylphenol). The extracted carotenoids were eluted at a flow rate of 0.8 mL/min with the following process: 100% A for 3 min; a liner gradient from 0 to 70% B within 2 min; a liner gradient from 70% B to 95% B within 4 min; a liner gradient from 95% B to 0 within 1 min; 100% A for 1 min. The injection volume was 2 μL. The effluent from the column was directed to the Atmospheric Pressure Chemical Ionization (APCI) source interfaced to the Q-TOF mass spectrometer. The ionization parameters were set with a temperature of 350 °C and a nebulizer pressure of 25 psi. The absorption spectra, retention times, and peak area of each carotenoid were compared with standards. The standard curve linear equations and correlation coefficients of all standards are listed in Table S3. The Bacterial Carotenoid Content (BCC) was calculated with the formula:

BCC (μg/g) = C × V/1000/M

where C indicates the concentration value obtained by substituting the integral peak area in the sample into the standard curve (μg/mL); V indicates the volume of the solution used for redissolution (μL); M indicates the sample quality (g).

#### 4.4.2. Determination of Total Carotenoid Content in Bacterial Culture Medium

During the cultivation period, three parallel samples were collected at 24 and 48 h for the determination of carotenoid content. Samples were mixed and centrifuged at 6000 r/min for 10 min at 4 °C. To ensure thorough washing, 20 mL of culture was rinsed twice with 50 mmol phosphate buffer (pH 7.0), followed by a repeat of the centrifugation process. Each freeze-dried bacterial sample was ground into a fine powder using a mortar and pestle. Subsequently, each powdered sample was combined with 10 mL mixed solution of hexane/acetone/ethanol (1:1:1, *v*/*v*/*v*) containing 0.01% 2-tert-butyl-4-methylphenol (BHT). This mixture was placed in an ice bath for ultrasonic treatment over 1 min. Finally, the supernatant was obtained by 8000 r/min centrifugation for 10 min at 4 °C. The supernatant was determined by measuring the absorbance at 449 nm. The Fermentation Broth Carotenoid (FBC) content was calculated with the formula:

FBC (mg/L) = A D V_1_/0.16 V_2_

where A indicates the supernatant with measured absorbance at 449 nm; D indicates the dilution multiple; V_1_ indicates the total volume of the extraction solution; 0.16 indicates the uniform extinction coefficient of carotenoids; V_2_ indicates the volume of the fermentation broth.

### 4.5. Data Processing

Raw data files acquired by LC-MS analysis were first converted to the “mzXML” format using the ProteoWizard software (https://proteowizard.sourceforge.io/, accessed on 1 April 2023) and then subjected to peak extraction, peak alignment, and retention time correction using XC-MS software (v. 3.5.1, Scripps Research Institute, La Jolla, CA, USA). The peak areas were corrected by the “SVR” method, and the peaks with a missing rate more than 50% were filtered in each group. The corrected and screened mass spectrometry peaks were used for compound identification based on retention time, secondary fragment ion peaks, and accurate mass numbers. The compared mass spectrometry databases included self-built database (Metware Biotechnology Co., Ltd., Wuhan, China), NIST (https://www.nist.gov/data, accessed on 4 May 2023), and METLIN (https://metlin.scripps.edu, accessed on 4 May 2023) database. The mass spectrometry multiple reaction monitoring (MRM) mode was employed for the quantification of the identified compounds. The raw data were centralized and processed using the MetaboAnalystR package in R software (https://rdrr.io/github/xia-lab/MetaboAnalystR3.0/man/, accessed on 4 May 2023) to perform OPLS-DA model calculation and analysis using the OPLSR.Anal function. Differential metabolites and intermediates in the biosynthesis of terpenoids were annotated using the KEGG metabolic pathway database.

Using Analyst 1.6.3 software to process mass spectrometry data, the total ion flow chart and extracted ion flow chart were obtained. Mass spectrometry data were processed using MultiQuant 3.0.3 software. The qualitative analysis of carotenoids in the samples was conducted by comparing the retention time and peak shape with those of standard samples. To ensure the accuracy of sample quantification, the chromatographic peaks of the analyte were integrated and corrected in the multiple reaction detection mode of triple quadrupole mass spectrometry. 

## 5. Conclusions

In this study, we introduce the inaugural metabolomic analysis of *Sphingopyxis*, successfully pinpointing a multitude of key metabolites essential to the biosynthesis of carotenoids through an exhaustive examination of the bacterium’s metabolic profile. We have systematically delineated the synthesis pathway of carotenoids: fructose is metabolized via glycolysis to produce glyceraldehyde 3-phosphate and pyruvate. These intermediates then serve as precursors for the synthesis of GFDP via MEP pathway. Subsequently, GFDP undergoes a cascade of reactions, including condensation, dehydrogenation, cyclization, and hydroxylation, along the carotenoid biosynthetic pathway, ultimately leading to the formation of various xanthophyll compounds. The strain USTB-05 grew with glucose as the carbon source, beef paste as the nitrogen source, a carbon to nitrogen ratio of 5, and an initial pH of 7.2. Under these conditions, the strain USTB-05 achieved a carotenoid concentration of 3.3 mg/L after a 48 h cultivation period. This research on the biosynthesis of carotenoids, encompassing both metabolic and cultural aspects, holds not only fundamental scientific value, but also significant potential for practical applications.

## Figures and Tables

**Table 1:** Classification and number of metabolites identified in strain USTB-05.

Number	Category	Negative Ion Mode	Positive Ion Mode	Total
1	Amino acid and its metabolites	360	564	924
2	Benzene and substituted derivatives	609	464	1073
3	Heterocyclic compounds	469	449	918
4	Organic acid and its derivatives	360	224	584
5	Aldehyde, ketones, esters	237	253	490
6	Alcohol and amines	142	156	298
7	Glycerophospholipids	71	121	192
8	Fatty acids	87	104	191
9	Nucleotide and its metabolites	100	66	166
10	Hormones and hormone related compounds	44	52	96
11	Flavonoids	50	32	82
12	Terpenoids	36	49	85
13	Alkaloids	35	45	80
14	Carbohydrates and its metabolites	60	13	73
15	Glycerolipids	7	32	39
16	CoEnzyme and vitamins	10	23	33
17	Lignans and coumarins	13	14	27
18	Sphingolipids	6	17	23
19	Steroids	7	13	20
20	Tryptamines, cholines, pigments	9	10	19
21	Bile acids	7	9	16
22	Phenolic acids	7	0	7
23	Quinones	2	1	3
24	Tannins	0	1	1
25	Others	224	217	441

**Table 2:** Identified carotenoids of strain USTB-05 based on LC-MS analysis.

Type	Category	R.T	Mother Ion Molecular Weight (Da)	Characteristic Fragment Ion Molecular Weight (Da)	Molecular Weight (Da)
α-carotene	carotenes	5.93	537.5	123.2	536.4382
β-carotene	carotenes	6.29	537.6	177.1	536.4382
phytoene	carotenes	4.98	545.3	81.0	544.5008
ε-carotene	carotenes	5.53	537.6	123.2	536.4382
phytofluene	carotenes	1.91	543.5	81.2	542.4852
γ-carotene	carotenes	7.40	537.4	177.3	536.4382
lycopene	carotenes	8.35	537.4	81.0	536.4382
echinenone	xanthophylls	5.55	551.6	203.1	550.9000
zeaxanthin	xanthophylls	4.64	569.4	477.5	568.4280
violaxanthin	xanthophylls	1.59	601.4	221.0	600.4179
β-cryptoxanthin	xanthophylls	5.53	553.5	177.4	552.4331
β-citraurin	xanthophylls	2.79	433.3	341.1	432.6000
astaxanthin	xanthophylls	3.42	597.3	147.1	596.8400

**Table 3:** The content and changes of the main synthetic carotenoids in strain USTB-05.

Type	Category	Molecular Formula	Cultivation 24 h (μg/g)	Cultivation 48 h (μg/g)	Change Amount (%)
α-carotene	carotenes	C_40_H_56_	0.3 ± 0.03 ^a^	0.23 ± 0.01 ^b^	−23.21
β-carotene	carotenes	C_40_H_56_	0.14 ± 0.02 ^a^	0.02 ± 0.01 ^b^	−84.46
phytoene	carotenes	C_40_H_64_	2.32 ± 0.24 ^a^	0.35 ± 0.18 ^b^	−84.85
phytofluene	carotenes	C_40_H_62_	0.5 ± 0.04 ^a^	0.42 ± 0.03 ^b^	−17.34
zeaxanthin	xanthophylls	C_40_H_56_O_2_	37.06 ± 0.81 ^a^	35.25 ± 0.79 ^b^	−4.9

Note that all values are the average values of three different samples, with different letters in the same row indicating the degree of difference (*p* < 0.05), and the “−” in front of the numbers indicate decreasing variations.

## Data Availability

Data are contained within the article.

## Supplementary Materials

The following supporting information can be downloaded at: https://www.mdpi.com/article/10.3390/molecules29174235/s1, Figure S1: TIC of metabolite profiles of methanol aqueous solution extracts from freeze-dried bacterial cells acquired in ESI(+) and ESI(−). The figure shows the results of three repeated experiments. Figure S2: TIC of carotenoid chromatograms of the organic solvent extracts from freeze-dried bacterial cells. Table S1. Retention time (Rt, min), proposed formula, molecular weight (Da), parent ion (Da), mass error (ppm), adduct ion of compounds identified in bacterial cells extracts with the use of UPLC-Q-TOF. Table S2. Retention time (Rt, min), proposed formula, molecular weight (Da), parent ion (Da), mass error (ppm), adduct ion of compounds identified carotenoid biosynthetic pathways in bacterial cells extracts with the use of UPLC-Q-TOF. Table S3. Linear equation and correlation coefficient of the standard curve for detected carotenoids.
