# Supplementary material for: Metabolomic Analysis of Carotenoids Biosynthesis by Sphingopyxis sp. USTB-05
Source: Molecules. 2024 Sep 6;29(17):4235. doi: 10.3390/molecules29174235 (PMC11397044; doi:10.3390/molecules29174235)
Supplement: Supplementary file 1 [file molecules-29-04235-s001.zip › Supplementary Materials.pdf]

Supplementary Figures and Tables

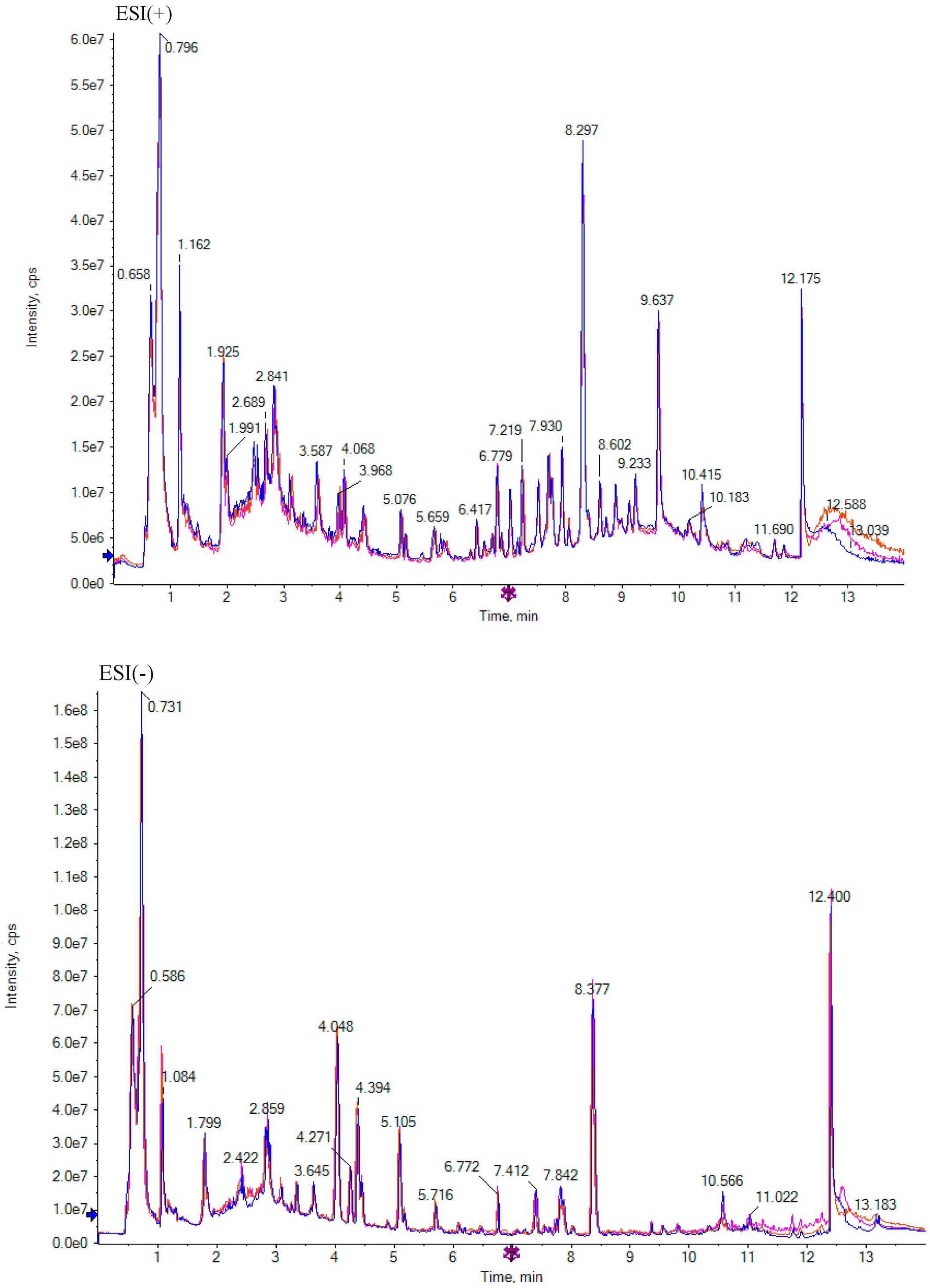

Figure S1: TIC of metabolite profiles of methanol aqueous solution extracts from freeze-dried bacterial cells acquired in ESI(+) and ESI(−). The figure shows the results of three repeated experiments.

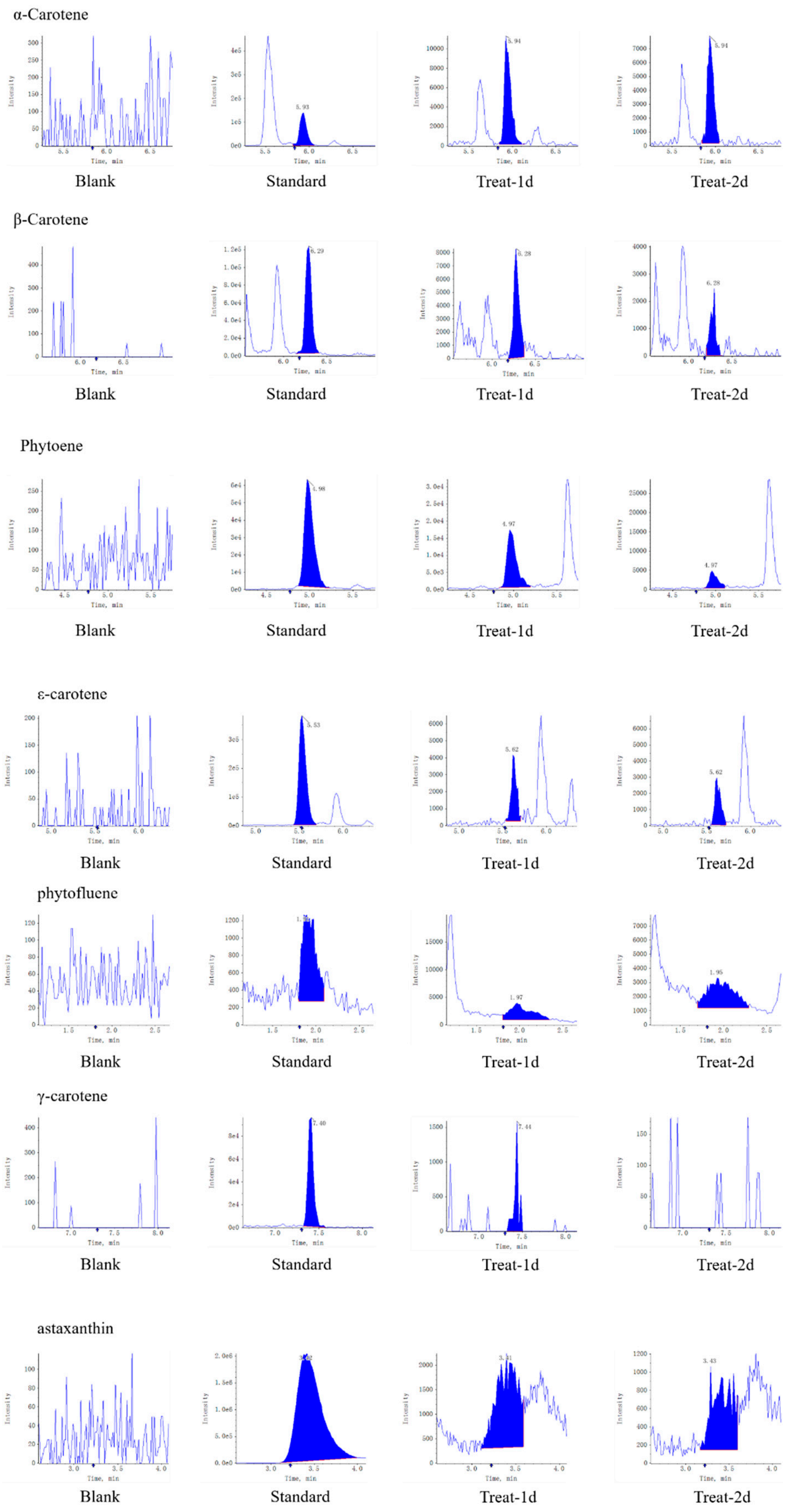

α-Carotene
Blank
Standard
Treat-1d
Treat-2d
β-Carotene
Blank
Standard
Treat-1d
Treat-2d
Phytoene
Blank
Standard
Treat-1d
Treat-2d
ε-carotene
Blank
Standard
Treat-1d
Treat-2d
phytofluene
Blank
Standard
Treat-1d
Treat-2d
γ-carotene
Blank
Standard
Treat-1d
Treat-2d
astaxanthin
Blank
Standard
Treat-1d
Treat-2d

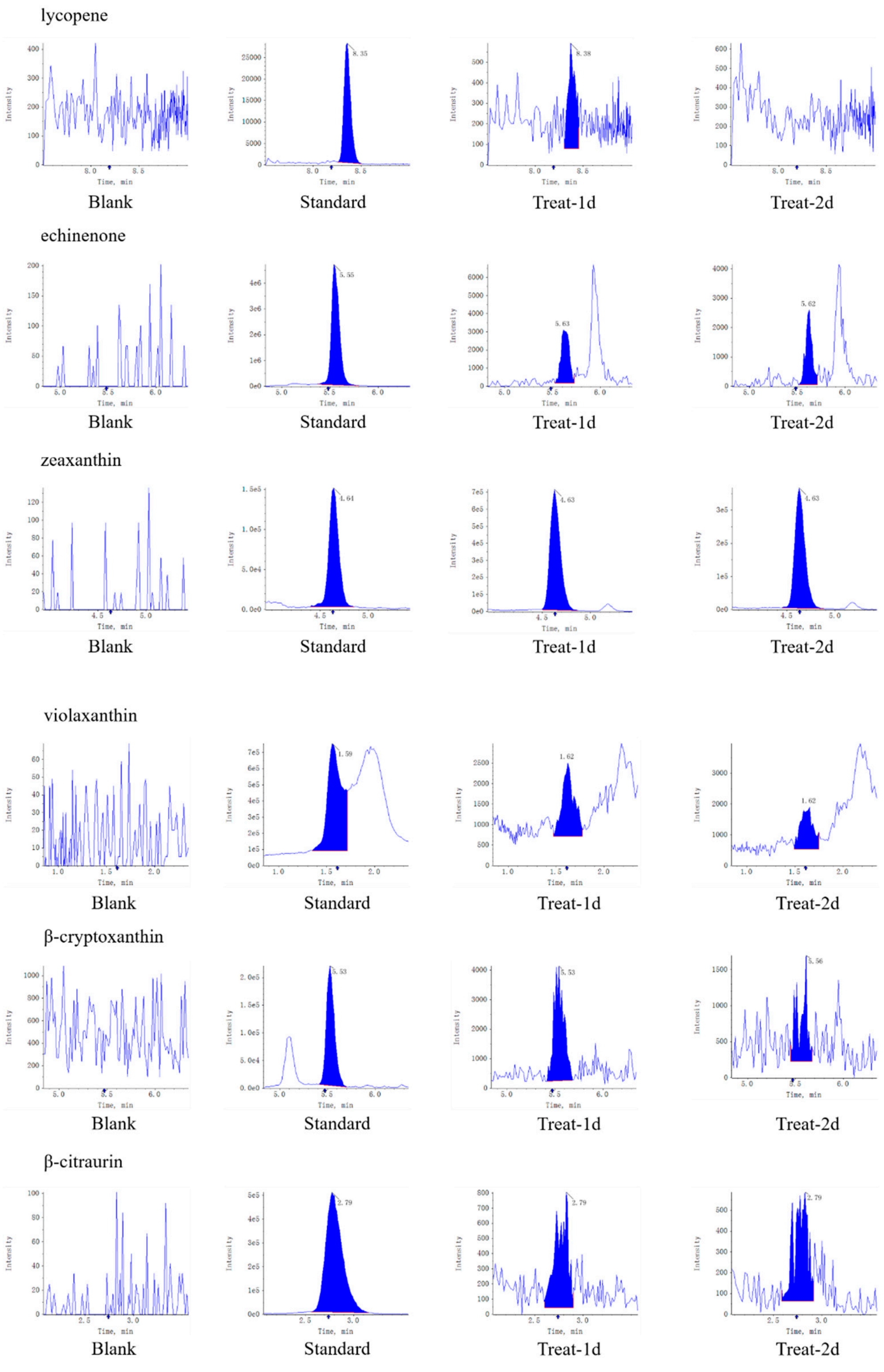

Figure S2: TIC of carotenoid chromatograms of the organic solvent extracts from freeze-dried bacterial cells.

Table S2. Retention time (Rt, min), proposed formula, molecular weight (Da), parent ion (Da), mass error (ppm), adduct ion of compounds identified carotenoid biosynthetic pathways in bacterial cells extracts with the use of UPLC-Q-TOF.

No	Rt [min]	Compound	Formula	Molecular weight [Da]	Parent ion [Da]	Mass error [ppm]	Adduct ion	BP
1	0.69	Fructose	$C_6H_{12}O_6$	180.0634	215.0337	2.57	M+Cl	G
2	1.05	Fructose 6-Phosphate	$C_6H_{13}O_9P$	260.0297	259.0217	0.38	M-H	G
3	12.66	Fructose-1,6-Biphosphate-Trisodium Salt	$C_6H_{11}Na_3O_{12}P_2$	405.9419	404.9395	12.62	M+3Na-H	G
4	2.3	2-Deoxyribose 5-Triphosphate	$C_5H_{13}O_{13}P_3$	373.9569	372.9424	16.96	M-H	G
5	0.67	3-Phosphoglycerate	$C_3H_7O_7P$	185.9929	186.9951	13.97	M+H	G
6	0.97	Phosphoenolpyruvate	$C_3H_5O_6P$	167.9824	226.9962	0.03	M+CH_3COO	G
7	2.97	Acetyl CoA	$C_{23}H_{38}N_7O_{17}P_3S$	809.1258	810.1335	0.53	M+H	Mv
8	0.82	Mevalonate	$C_6H_{12}O_4$	148.0736	188.0927	0.16	M-H+CH_3CN	Mv
9	1.05	Mevalonate 5-Phosphate	$C_6H_{13}O_7P$	228.0399	273.0361	2.5	M+HCOO	Mv
10	1.04	D-Xylulose 5-Phosphate	$C_5H_{11}O_8P$	230.0192	289.0329	1.59	M+CH_3COO	Me
11	1.21	2-Methyl-D-Erythritol 2,4-Cyclodiphosphate	$C_5H_{12}O_9P_2$	277.9957	278.0024	14.77	M-	Me
12	5.26	3-Dimethylallyl-4-Hydroxybenzaldehyde	$C_{12}H_{14}O_2$	190.0994	235.0952	1.81	M+HCOO	Me
13	2.88	Isopentenyl Pyrophosphate	$C_5H_{12}O_7P_2$	246.0058	246.0981	19.44	M-	Me
14	4.15	Farnesyl Diphosphate	$C_{15}H_{28}O_7P_2$	382.131	427.1293	1.27	M+HCOO	Me
15	9.37	Heptaprenyl Diphosphate	$C_{35}H_{57}O_7P_2$	651.358	650.3645	29.24	M-H	Me
16	0.74	Citrate	$C_6H_5O_7$	189.0035	173.0089	0.5	M-H_2O-H	T
17	1.07	Isocitric	$C_6H_8O_7$	192.027	173.0088	0.75	M-H_2O-H	T
18	9.63	N-Methyl-2-Oxoglutaramic Acid	$C_6H_9NO_4$	159.0532	223.0636	13.4	M+CH_3CN+Na	T
19	7.71	Methylprednisolone Sodium Succinate	$C_{26}H_{33}NaO_8$	496.2073	519.1971	4.66	M+Na	T
20	6.38	Geranylfarnesyl Diphosphate	$C_{25}H_{41}O_7P_2$	515.2328	618.2009	17.95	M+Cl+NaCOOH	C
21	10.61	Phytoene	$C_{40}H_{64}$	544.5008	544.506	10.63	M+	C
22	10.75	Dihydroxy-β-Carotene	$C_{40}H_{58}O_2$	570.4437	571.4609	16.98	M+H	C
23	6.01	Echinenone	$C_{40}H_{54}O$	550.4175	589.3863	8.24	M+K	C
24	3.94	Hydroxyastaxanthin	$C_{40}H_{52}O_5$	612.3815	583.3814	12.21	M-H-CO	C

Biosynthetic pathways involved: G, glycolysis/gluconeogenesis pathway; Me, 2-C-methyl-D-erythritol 4-phosphate pathway; Mv, mevalonic acid pathway; T, tricarboxylic acid cycle; C, carotenoid biosynthesis pathway.

Table S3. Linear equation and correlation coefficient of the standard curve for detected carotenoids.

No	Compounds	Retention time	Linear equation	Correlation coefficient
1	α-carotene	5.93	y = 3.46281e5 x + 1083.99781	0.99458
2	lycopene	8.35	y = 1.17062e5 x - 569.47533	0.9916
3	γ-carotene	7.40	y = 5.65435e5 x - 18140.05327	0.99127
4	β-carotene	6.29	y = 6.91386e5 x - 1853.60233	0.99463
5	phytofluene	1.91	y = 2.26494e5 x - 801.01114	0.99557
6	phytoene	4.98	y = 1.03263e5 x + 129.86713	0.99666
7	ε-carotene	5.53	y = 1.21098e6 x - 1467.90989	0.99413
8	violaxanthin	1.59	y = 3.24166e6 x - 980.48623	0.99438
9	zeaxanthin	4.64	y = 2.99149e5 x - 731.12382	0.99627
10	β-cryptoxanthin	5.53	y = 4.84221e5 x – 1428.27343	0.99226
11	astaxanthin	3.42	y = 3.20467e6 x – 566.00547	0.9941
12	echinenone	5.55	y = 1.69030e7 x - 1226.13336	0.99548
13	β-citraurin	2.79	y = 5.43472e6 x - 380.88748	0.99233
